# Metabolic and vascular imaging markers for investigating Alzheimer’s disease complicated by sleep fragmentation in mice

**DOI:** 10.3389/fphys.2024.1456690

**Published:** 2024-09-20

**Authors:** Xiaoning Han, Guanshu Liu, Sang Soo Lee, Xiuli Yang, Mark N. Wu, Hanzhang Lu, Zhiliang Wei

**Affiliations:** ^1^ Department of Anesthesiology and Critical Care Medicine, Johns Hopkins University School of Medicine, Baltimore, MD, United States; ^2^ Russell H. Morgan Department of Radiology and Radiological Science, Johns Hopkins University School of Medicine, Baltimore, MD, United States; ^3^ F. M. Kirby Research Center for Functional Brain Imaging, Kennedy Krieger Research Institute, Baltimore, MD, United States; ^4^ Department of Neurology, Brain Science Institute, Johns Hopkins University School of Medicine, Baltimore, MD, United States; ^5^ Department of Biomedical Engineering, Johns Hopkins University School of Medicine, Baltimore, MD, United States

**Keywords:** Alzheimer’s disease, sleep fragmentation, oxygen extraction fraction, cerebral blood flow, cerebral metabolic rate of oxygen, neuroinflammation, 5xFAD

## Abstract

**Background:**

Sleep problem is a common complication of Alzheimer’s disease (AD). Extensive preclinical studies have been performed to investigate the AD pathology. However, the pathophysiological consequence of AD complicated by sleep problem remains to be further determined.

**Purpose:**

To investigate brain metabolism and perfusion in an AD mouse model complicated by sleep problem, and subsequently identify potential imaging markers to better understand the associated pathophysiology.

**Methods:**

We examined the oxygen extraction fraction (OEF), cerebral metabolic rate of oxygen (CMRO_2_), and cerebral blood flow (CBF) using state-of-the-art MRI techniques in a cohort of 5xFAD model mice. Additionally, neuroinflammation, indicated by activated microglia, was assessed using histology techniques. Sleep fragmentation (SF) was utilized as a representative for sleep problems.

**Results:**

SF was associated with significant increases in OEF (*P* = 0.023) and CMRO_2_ (*P* = 0.029), indicating a state of hypermetabolism. CBF showed a significant genotype-by-sleep interaction effect (*P* = 0.026), particularly in the deep brain regions such as the hippocampus and thalamus. Neuroinflammation was primarily driven by genotype rather than SF, especially in regions with significant interaction effect in CBF measurements.

**Conclusion:**

These results suggest that brain metabolism and perfusion measurements are promising markers for studying the co-pathogenesis of AD and SF.

## 1 Introduction

Sleep is vital for maintaining health in various aspects, e.g., resetting the immune system, restoring hormonal balance, and clearing metabolic wastes and neurotoxins (Besedovsky et al., 2019; Kim et al., 2015; Reddy and van der Werf, 2020). Unfortunately, over 30% of Americans experience sleep disruptions to varying extents due to insufficient sleep time or poor sleep quality (Yong et al., 2017). Compared with the younger population, older adults exhibit age-dependent sleep changes, including decreased slow-wave sleep, sleep fragmentation (SF), and early awakening (Carskadon et al., 1982; Patel et al., 2018). Short-term sleep disturbances lasting a few days can disrupt emotions and cognition, and are generally reversible after weeks of good rest (Ochab et al., 2021; Kitamura et al., 2016). However, long-term chronic sleep disturbances can introduce lasting detrimental effects on neurobehavioral performances and lead to cardiovascular diseases, weight-related issues, metabolic syndromes, and type-II diabetes (Sabanayagam and Shankar, 2010; Kline et al., 2021; Peila et al., 2022; Mehrdad et al., 2022).

Epidemiological studies have revealed that 25%–44% of dementia patients suffer from sleep disruptions (Moran et al., 2005; Zhao et al., 2016; Vitiello and Borson, 2001). Persistent sleep disruption between the ages of 50–70 is associated with a 30% increased risk of dementia, independent of sociodemographic, behavioral, cardiometabolic, and mental health factors (Sabia et al., 2021). Moreover, it has been found that sleep disruption accelerates the accumulation of Aβ and tau, contributing to the pathological progression of Alzheimer’s disease (AD) and impairing cognitive functions (Holth et al., 2019; Tabuchi et al., 2015; Holth et al., 2017). Currently, sleep disruption is often considered a risk factor for AD development. SF, which refers to transient arousals with short-interval awakenings and sleep-related respiration disturbances during sleep, is a subtype of sleep disruption with significant clinic relevance, as individuals with AD experience more fragmented sleep (Carskadon et al., 1982; Vanderheyden et al., 2018). Given the prevalence of sleep problems in various types of dementia and psychiatric conditions, the interplay between sleep perturbation and neurodegenerative pathology has attracted broad attentions.

The pathological progression of AD is known to be progressive (Abubakar et al., 2022). Early biomarkers are crucial for identifying vulnerable populations, diagnosing patients, and monitoring the efficacy of interventions or treatments. Cerebral physiology provides a rich source of biomarkers for AD (Goldsmith, 2022; Korte et al., 2020; Lin Z. et al., 2019; Thomas et al., 2017; Lin et al., 2021; Sur et al., 2020; Harrison et al., 2020), including brain perfusion (Goldsmith, 2022; Korte et al., 2020) and metabolism (Lin Z. et al., 2019; Thomas et al., 2017), which have shown feasibility in both animal models and human studies of AD pathology (Wei et al., 2021; Lu et al., 2011). Reduced brain metabolism was found in the human patients of mild cognitive impairment (MCI), an early form of AD (Thomas et al., 2017). Such a cerebral hypometabolism was confirmed with AD mouse models and was primarily linked to the neurodegeneration (Wei et al., 2021). Meanwhile, the spatial-temporal pattern of brain perfusion loss from precuneus, posterior cingulate and temporal-parietal regions to broader areas was revealed during the pathological progression from healthy control to MCI to AD, supporting the incorporation of brain perfusion into the AD research framework (Zhang et al., 2021). On the other hand, a neuroimmune axis was identified linking sleep to atherosclerosis (McAlpine et al., 2019), which is a common vascular dysfunction. Different patterns of regional hypoperfusion were distinctively associated with SF (Yan et al., 2021). Based on these evidences, microvascular dysfunction plays an important role in the pathological progression of AD or SF. While the validation of physiological markers in single AD or SF pathology has been extensively reported (Thomas et al., 2017; Zhang et al., 2021; Yan et al., 2021), parallel studies focusing on the co-pathogenesis of AD and SF has been limited. Therefore, this study aims to fill this knowledge gap by characterizing the cerebral metabolic and vascular profiles with non-contrast MRI techniques in a mouse model of AD complicated by SF. The mouse model utilized is the 5xFAD (Oakley et al., 2006), an amyloidosis model that begins to exhibit extracellular amyloid deposition around 2 months of age, initially in the cortex and subsequently throughout the hippocampus. Cerebral physiological parameters of oxygen extraction fraction (OEF), cerebral blood flow (CBF), and cerebral metabolic rate of oxygen (CMRO_2_) will be investigated, along with neuroinflammation assessed by activated microglia.

## 2 Methods

### 2.1 General

**FIGURE 1:**
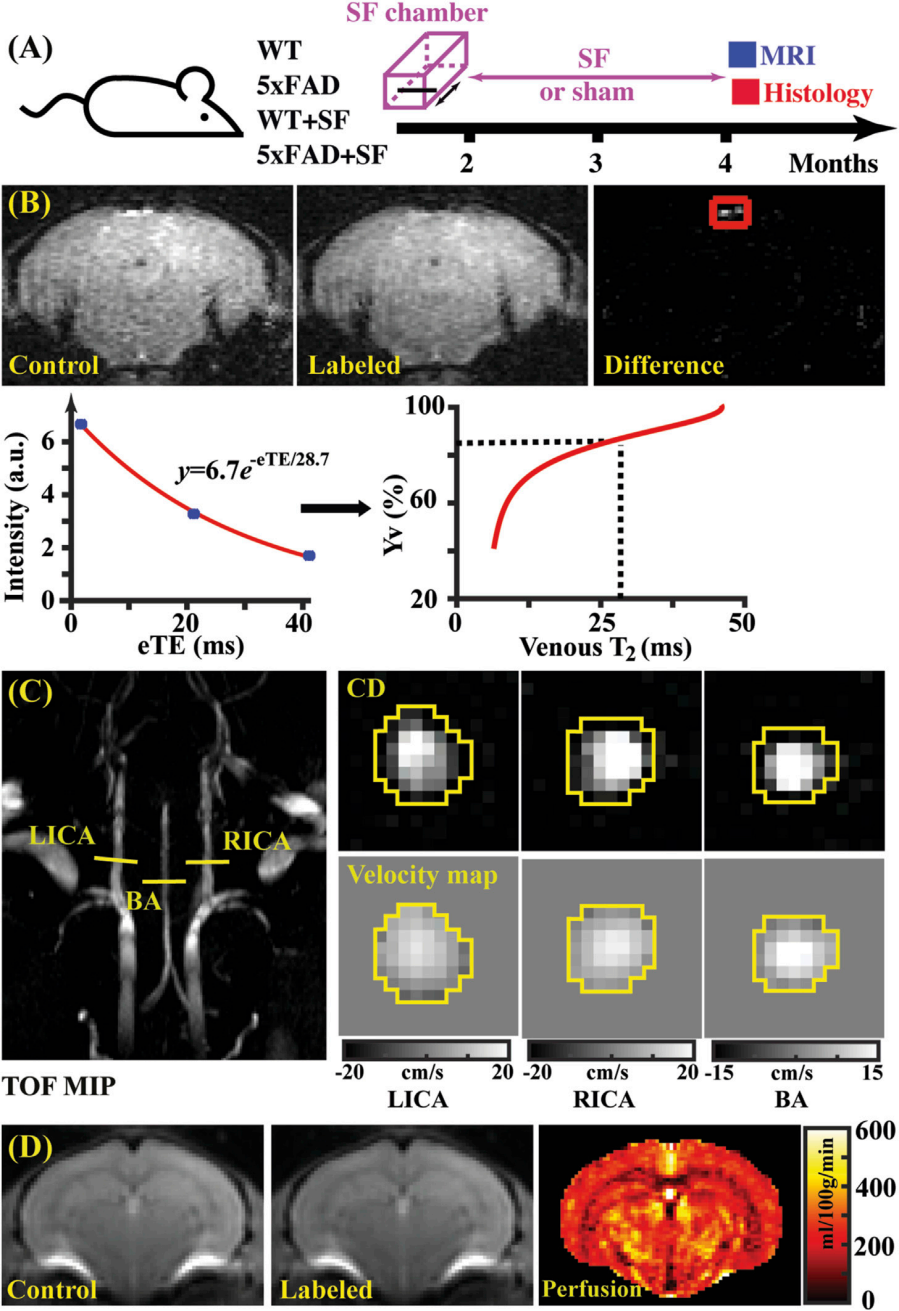
Study design **(A)** and representative images of TRUST **(B)**, PC **(C)**, and pCASL **(D)** MRI. **(B)** Top panel: control, labeled, and difference images for TRUST obtained at the eTE of 0.25 ms. Sinus confluence was marked by a red square. Bottom panel: signal intensities of venous blood as a function of eTE. The fitted venous T_2_ can be converted into oxygenation by reference to the T_2_-Yv calibration plot (Li and van Zijl, 2020). **(C)** Left panel: maximal intensity project (MIP) of time-of-flight (TOF) MRI to visualize the feeding arteries. Right panel: complex difference (CD) images and velocity maps for the three feeding arteries (LICA, RICA, and BA). **(D)** Control, labeled, and perfusion images from the pCASL MRI.

The experimental protocols for this study were approved by the Johns Hopkins Medical Institution Animal Care and Use Committee and conducted in accordance with the National Institutes of Health guidelines for the care and use of laboratory animals. According to the pathological cascade model of AD, amyloid β (Aβ) deposition is an earlier event than tau-mediated neuronal injury and dysfunction, brain structure change, and memory decline (Jack et al., 2013). Therefore, the popularly utilized 5xFAD model (Oakley et al., 2006), which is an amyloidosis model expressing human APP and PSEN1 transgenes, was selected and studied. A total of 24 male mice in the C57BL/6 background were used in this study, including 12 5xFAD (MMRRC-034848-JAX) (Oakley et al., 2006) and 12 littermate wildtype (WT) mice. These mice were further divided into subgroups with equal mouse numbers to undergo either SF or sham operations, resulting in four experimental groups: WT (N = 6), 5xFAD (N = 6), WT + SF (N = 6), and 5xFAD + SF (N = 6). SF was induced using a previously reported protocol (McAlpine et al., 2019) from 2 to 4 months old with specially designed chambers (Figure 1A). Using identical procedures, biochemical evaluations including hypocretin changed significantly after 12 weeks but not 6 weeks of SF (McAlpine et al., 2019). Therefore, in this study, we selected 8 weeks of SF as a compromise to track observable pathological changes while identifying early functional marker(s). In brief, mice were placed in the SF chamber (Lafayette Instrument) with a sweeping bar moving along the bottom every 2 min during the light cycle (Zeitgeber time, ZT; ZT0-ZT12). Sham operation was keeping mice in the SF chamber without sleep interruption. External environment for the chambers was a quiet local animal facility with 12 h day/night cycle and all mice had free access to food and water. Mice losing more than 10% of their original body weights or showing signs of significant stress would be excluded from experiments and analyses. At the time of MRI scans, there was no significant difference in body weight among mice in different experimental groups (ANOVA, *P* = 0.41).

### 2.2 MRI

All MRI experiments were conducted on an 11.7T Bruker Biospec system (Bruker, Ettlingen, Germany) equipped with a horizontal bore and actively shielded pulsed field gradients (maximum intensity of 0.74 T/m). Images were acquired using a 72-mm quadrature volume resonator as the transmitter and a four-element (2 × 2) phased-array coil as the receiver. B_0_ homogeneity across the mouse brain was optimized using global shimming (up to 2nd order) based on a pre-acquired subject-specific field map.

Respiration rate was monitored during the experiment using a MR-compatible monitoring and gating system (SA Instruments). To measure heart rate, an MRI sequence known as ultrashort TE (UTE) MRI was utilized. This sequence repeatedly acquired the center *k*-space every 8.0 ms, producing a time course of MR signal intensity where the period corresponded to the R-R interval. Anesthesia was administered according to the following regimen: 1.5% vaporized isoflurane was administered for 15 min as an induction followed by continuous 1.0% isoflurane for maintenance until the end of experiments; during the experiment, the maintenance dosage would be increased slightly to ∼1.2% if a mouse exhibited respiration rates exceeding 150 breath/min. At the 10th minute under 1.5% isoflurane inhalation, mouse was immobilized using a bite bar and ear pins, and then positioned on a water-heated animal bed with temperature control before entering the magnet.

The order of MRI scans for each mouse was randomized using the following scheme: initially, each mouse was preassigned to consecutive numbers from one. Using the MATLAB (MathWorks, Natick, United States) “rand” function, an array of pseudorandom numbers was generated. The ranks of these numbers, sorted from largest to smallest, determined the order of experimental scans. For example, if the first pseudorandom number ranked third in the array, the mouse preassigned to number 3 would be scanned first (Wei et al., 2021). Each MRI session included the following detailed measurements.

#### 2.2.1 Brain volume

A T_2_-weighted fast spin-echo MRI protocol was utilized to collect anatomical MRI images for estimating brain volume. The imaging parameters were: repetition time (TR) = 3,000 ms, time to echo (TE) = 11 ms, field of view (FOV) = 15 mm × 15 mm, matrix size = 128 × 128, slice thickness = 0.5 mm (without inter-slice gap), echo spacing = 5.5 ms (4 spin echoes per scan), 35 axial slices, and scan duration = 1.6 min (Wei et al., 2021).

Subsequently, the T_2_-weighted images were analyzed manually by delineating the brain boundary on a slice-by-slice basis, referencing to a mouse brain atlas (https://atlas.brain-map.org/). Voxel counts within the delineated masks were summed to calculate the total brain volume in cm^3^.

#### 2.2.2 Oxygen extraction fraction (OEF)

OEF is defined as the arteriovenous oxygenation difference, i.e., $OEF=Y_{a}-Y_{v}$ , where Y_a_ denotes arterial oxygenation and is assumed to be 0.99 (Wei et al., 2020) and Y_v_ denotes venous oxygenation assessed by the T_2_-relaxation-under-spin-tagging (TRUST) technique (Wei et al., 2018). TRUST was originally developed for human scanners (Lu and Ge, 2008) and then optimized for animal MRI systems (Wei et al., 2018; Xu et al., 2021). Imaging slice was positioned to cover the confluences of sagittal sinuses (Wei et al., 2018). In order to visualize the confluence of sagittal sinuses, an axial time-of-flight (TOF) sequence was performed with the following parameters: TR/TE = 20/2.6 ms, FOV = 16 mm × 16 mm, matrix size = 256 × 256, 35 axial slices, slice thickness = 0.5 mm, and scan duration = 2.2 min. The TRUST scan was repeated twice to enhance measurement fidelity with the following parameters: TR/TE = 3,500/6.5 ms, FOV = 16 mm × 16 mm, matrix size = 128 × 128, slice thickness = 0.5 mm, echo-planar imaging (EPI) acquisition factor = 16, inversion-slab thickness = 2.5 mm, post-labeling delay = 1,000 ms, effective echo time (eTE) = 0.25, 20, 40 ms, echo spacing of eTE = 5.0 ms, and scan duration = 2.8 min.

Data processing of TRUST MRI was conducted with a custom-written graphic-user-interface (GUI) tool built on MATLAB (MathWorks, Natick, MA) and followed procedures detailed previously (Wei et al., 2021; Wei et al., 2018). Briefly, for each TRUST dataset, subtraction between the control and labeled images was performed to obtain difference images (Figure 1B). A region of interest (ROI) was manually drawn on the difference image to encompass the confluence of sinuses. Four voxels within the ROI with the largest difference signals were automatically selected for spatial averaging. Then, venous blood signal intensities at three different eTEs were fitted into a monoexponential function to obtain T_2_ (Figure 1B). Finally, T_2_ was converted into Y_v_ using a T_2_-Y_v_ calibration plot (Figure 1B) reported by Li and van Zijl (2020).

#### 2.2.3 Cerebral blood flow (CBF)

Global CBF was assessed using phase-contrast (PC) MRI, targeting the three major feeding arteries (Figure 1C): the left internal carotid artery (LICA), right internal carotid artery (RICA), and basilar artery (BA), in separate scans to collect corresponding through-plane velocity maps (Figure 1C) (Wei et al., 2019). Prior to the PC scans, we conducted a coronal TOF angiogram (7 slices, slice thickness = 0.5 mm, no inter-slice gap, TR/TE = 45/2.6 ms, scan duration = 2.0 min) to visualize the feeding arteries. Subsequently, a sagittal TOF scan (single slice, tilted to contain the target artery identified from coronal TOF images, thickness = 0.5 mm, TR/TE = 60/2.5 ms, scan duration = 0.4 min) was performed to visualize the in-plane trajectory of the targeted artery. Finally, PC MRI was positioned by reference to these TOF images (coronal and sagittal) and scanned with the following parameters: TR/TE = 15/3.2 ms, FOV = 15 mm × 15 mm, matrix size = 300 × 300, slice thickness = 0.5 mm, number of average = 4, dummy scan = 8, receiver bandwidth = 100 kHz, flip angle = 25°, partial Fourier acquisition factor = 0.7, and scan duration = 0.6 min.

Data processing of PC MRI utilized a custom-written graphic-user-interface (GUI) tool developed in MATLAB following the established procedures (Wei et al., 2019; Wei et al., 2023a). The artery of interest was first delineated manually on the complex-difference image (Figure 1C), which provided clear contrast between the vessel and surrounding tissue. This mask was then applied to the phase velocity map, and integration of arterial voxels yielded blood flow through the targeted artery in mL/min. Summing the blood flow values from the three major feeding arteries provided the total blood flow to brain. To normalize the brain-size differences and obtain unit-mass CBF values, the total blood flow was divided by the brain weight, which was calculated as the product of brain volume and brain tissue density (1.04 g/mL) (Bothe et al., 1984). The global CBF value was reported in the unit of milliliters per 100 g brain tissue per minute (mL/100 g/min).

#### 2.2.4 Cerebral metabolic rate of oxygen (CMRO_2_)

CMRO_2_ was computed from Y_v_ and CBF using the Fick principle, i.e., ${\text{CMRO}}_{2}=C_{a}\cdot OEF\cdot CBF$ , where C_a_ denoted the molar concentration of oxygen in a unit volume of blood and was assumed to be 882.1 µmol O_2_/100 mL blood based on previous literature (Ulatowski et al., 1999). CMRO_2_ was reported in the unit of µmol oxygen per 100 g brain tissue per min (µmol O_2_/100 g/min).

#### 2.2.5 Regional perfusion

A two-scan pseudo-continuous arterial spin labeling (pCASL) optimized to minimize the influence of magnetic field inhomogeneity was utilized (Hirschler et al., 2018). First, a pre-scan was performed to optimize the phases of labeling pulses in the control and labeled scans. Then, the scan focusing on regional perfusion was performed with the following parameters: (Wei et al., 2023b) TR/TE = 3,000/11.8 ms, labeling duration = 1,800 ms, FOV = 15 mm × 15 mm, matrix size = 96 × 96, slice thickness = 0.75 mm, labeling-pulse width = 0.4 ms, inter-labeling-pulse delay = 0.8 ms, flip angle of labeling pulse = 40°, post-labeling delay = 300 ms, two-segment spin-echo echo-planar-imaging acquisition, partial Fourier acquisition factor = 0.7, number of average = 25, and scan duration = 5.0 min.

Processing of pCASL data followed the reported procedures (Wei et al., 2021). Briefly, pair-wise subtraction between control and labeled images (i.e., $M_{ctr}-M_{lbl}$ ) were first applied to yield a difference image, which was then divided by a M_0_ image (obtained by scaling the control image) to provide a perfusion index image, i.e., $CBF_{index}=\frac{M_{ctr}-M_{lbl}}{M_{0}}$ . The perfusion index maps were co-registered and normalized to a mouse brain template (Meyer et al., 2017) and then resized to recover original acquisition resolutions. The normalized perfusion index maps were rescaled by reference to the global CBF (from PC MRI) to obtain absolute-value CBF, specifically, $CBF_{reg}=\frac{CBF_{index}}{Mean\left(CBF_{index}\right)}CBF_{PC}$ (Wei et al., 2021). Regional-of-interest (ROI) was drawn on the averaged control images to encompass isocortex, hippocampus, thalamus, hypothalamus, and striatum by reference to the mouse brain atlas (https://atlas.brain-map.org/). Voxel-wise CBF values within each ROI were averaged to estimate the corresponding perfusion levels.

### 2.3 Immunofluorescence staining

Mice were euthanized for histological analyses after MRI sessions. Mice were transcardially perfused with phosphate-buffered saline (PBS) followed by 4% paraformaldehyde (PFA) solution. Brains were extracted and fixed in 4% PFA overnight, and then were dehydrated in a 30% sucrose solution. Brain tissue was sectioned into 30 µm slices using a microtome (Leica, SM2010R). Neuroinflammation was evaluated by quantifying the percentage of activated microglia (Yeo et al., 2019; Marschallinger et al., 2020). For each mouse, three slides from frontal and parietal regions at similar cerebral positions were selected and stained. Primary antibodies included rabbit anti-Iba1 antibody (FUJIFILM, 019-19741, 1:500), rat anti-CD68 antibody (BIO-RAD, MCA 1957, 1:500), and Hoechst (Thermo Scientific, 62249, 1:1,500). Staining procedures followed previous reports (Wang et al., 2023; Liu et al., 2023; Liu et al., 2021). Microscope slides were examined with Zeiss confocal microscopes (Carl Zeiss NTS Ltd., Jena, Germany). Scanning was performed with the Z-stack and tile-scan mode to cover the whole slices. Microscope settings were optimized initially with brain slides randomly selected from various experimental groups, and subsequently applied to all brain slides. Representative slices from each experimental group were first checked at ×20 magnification to confirm staining quality, followed by scanning all slices at ×10 magnification for quantification.

Visualization and quantification of microscope images were conducted with ImageJ and MATALB. When displaying whole-slice microscope images, convolution-based smoothing was applied to enhance visibility of staining-positive signals. Quantification was based on the original microscope images.

### 2.4 Statistical analyses

All statistical analyses were performed with custom-built MATLAB (MathWorks, Natick, MA) scripts. Multivariate linear regression model (LRM) was employed to analyze the genotype effect and sleep manipulation effect in different physiological (OEF, CBF, and CMRO_2_) and histological (activated microglia) measurements. Respiration rate and heart rate were included as co-variates for the LRM analyses focusing on physiological measurements because these two basic vital signs were markers for harmonizing anesthetic effects (Wei et al., 2023a; Wei et al., 2024). After the analyses focusing on a single factor, LRM analyses were repeated by including an additional genotype-by-sleep-manipulation (i.e., genotype × sleep) term to confirm the existence of interaction effect. When presenting effects, 95% confidence intervals (CI) were described correspondingly. Pearson correlation analysis was performed between cerebral perfusion and neuroinflammation in isocortex, hippocampus, thalamus, hypothalamus, and striatum separately. Nominal measurement values were given in the format of mean ± standard deviation. A *P*-value of <0.05 was considered significant.

## 3 Results

Figure 1B shows representative data for TRUST MRI, consisting of control, labeled, and difference images at different eTE values (eTE = 0.25 ms as an example). Signal intensities of venous blood at different eTE values were fitted into a monoexponential function to estimate venous T_2_, which was subsequently converted into Y_v_ for estimating OEF. Figure 1C presents representative dataset for PC MRI covering the three major feeding arteries (LICA, RICA, and BA). Artery regions were delineated on the complex difference (CD) images (yellow polygons), which showed clear contrast between arteries and surrounding tissues, and subsequently applied to the velocity map to quantify blood flow. Figure 1D shows a representative pCASL MRI data, which revealed cerebral perfusion with the difference signal between the control and labeled images.

**FIGURE 2:**
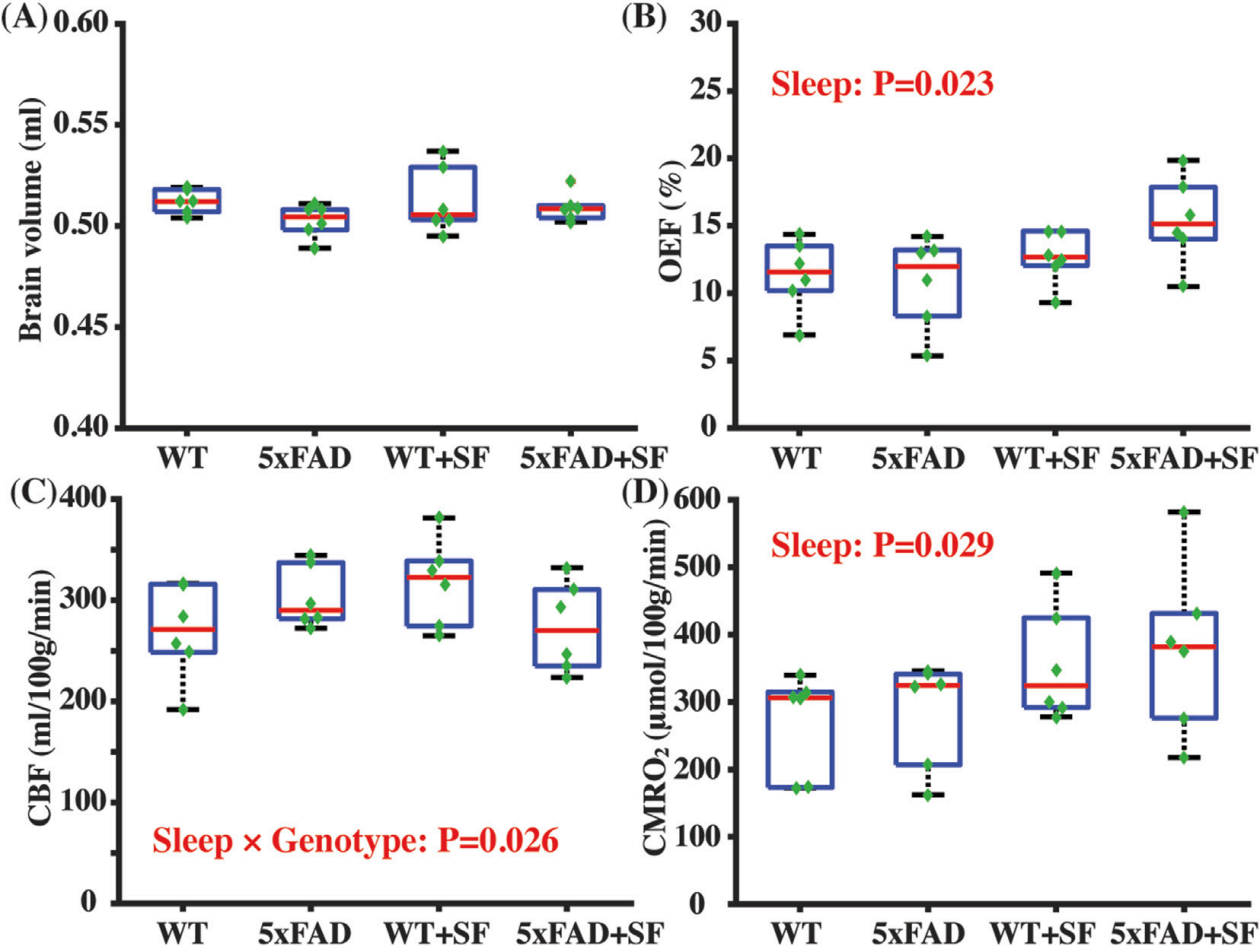
Metabolic and vascular alternation in 5xFAD complicated by SF. Comparisons of brain volume **(A)**, OEF **(B)**, CBF **(C)**, and CMRO_2_ **(D)** among the WT, 5xFAD, WT + SF, and 5xFAD + SF groups. In the boxplot, central red mark was median, top and down edges of the box were 25th and 75th percentiles, and the whiskers extended to the minimal and maximal datapoints which were not considered to be outliers.

Figure 2 summarizes the physiological parameters related to the cerebral oxygen metabolism. Brain volumes were similar among the WT, 5xFAD, WT + SF, and 5xFAD + SF groups (genotype effect: *P* = 0.14; sleep effect: *P* = 0.40; sleep × genotype effect: *P* = 0.47) (Figure 2A), suggesting minimal volumetric alterations due to genotype or SF. OEF was associated with a significant sleep effect (coefficient = 2.83%, CI = [0.42, 5.24], *P* = 0.023) but not a genotype effect (*P* = 0.81) (Figure 2B). There was not a genotype (*P* = 0.79) or sleep effect (*P* = 0.71) in CBF (Figure 2C). CMRO_2_ was significantly affected by sleep (coefficient = 84.79 µmol/100 g/min, CI = [9.59, 159.99], *P* = 0.029) but not by genotype (*P* = 0.90). After including the sleep × genotype interaction term in the statistical models, there was a significant interaction effect for CBF (coefficient = −84.50 mL/100 g/min, CI = [−157.61, −11.39], *P* = 0.026) but not for OEF (*P* = 0.20) or CMRO_2_ (*P* = 0.92), suggesting that vascular function in WT and 5xFAD mice were differentially affected by SF.

**FIGURE 3:**
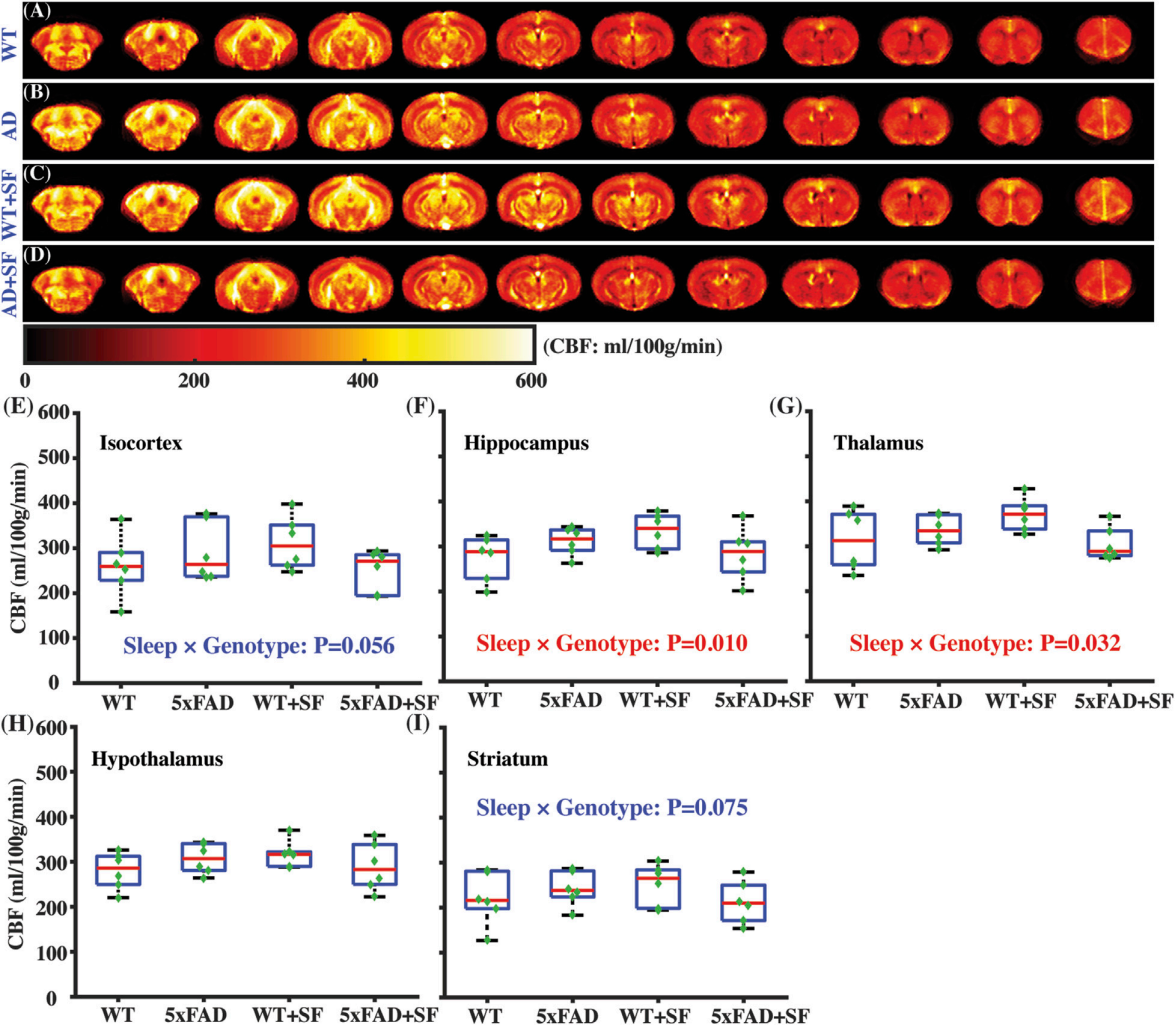
Regional perfusion alterations in 5xFAD complicated by SF. Averaged perfusion maps from caudal (left) to rostral (right) slices in WT **(A)**, 5xFAD **(B)**, WT + SF **(C)**, and 5xFAD + SF **(D)** by pCASL MRI. Perfusion comparisons in isocortex **(E)**, hippocampus **(F)**, thalamus **(G)**, hypothalamus **(H)**, and striatum **(I)**.

Figure 3 presents the regional perfusion alterations in the 5xFAD mice complicated by SF. Figures 3A–D show the averaged perfusion maps of WT, 5xFAD, WT + SF, and 5xFAD + SF groups. Overall data qualities were consistent across the four experimental groups. There was not a significant genotype effect (*P* ≥ 0.47) or sleep effect (*P* ≥ 0.54) in any of the investigated regions (isocortex, hippocampus, thalamus, hypothalamus, and striatum). The sleep × genotype interaction effect was significant for the perfusion in hippocampus (coefficient = −101.13 mL/100 g/min, CI = [−174.60, −27.67], *P* = 0.010; Figure 3F) and thalamus (coefficient = −90.35 mL/100 g/min, CI = [−171.76, −8.94], *P* = 0.032; Figure 3G), but not hypothalamus (*P* = 0.10; Figure 3H). Meanwhile, isocortex (coefficient = −100.15 mL/100 g/min, CI = [−203.05, 2.76], *P* = 0.056; Figure 3E) and striatum (coefficient = −72.01 mL/100 g/min, CI = [−151.99, 7.97], *P* = 0.075; Figure 3I) showed trends toward significant interaction effects.

**FIGURE 4:**
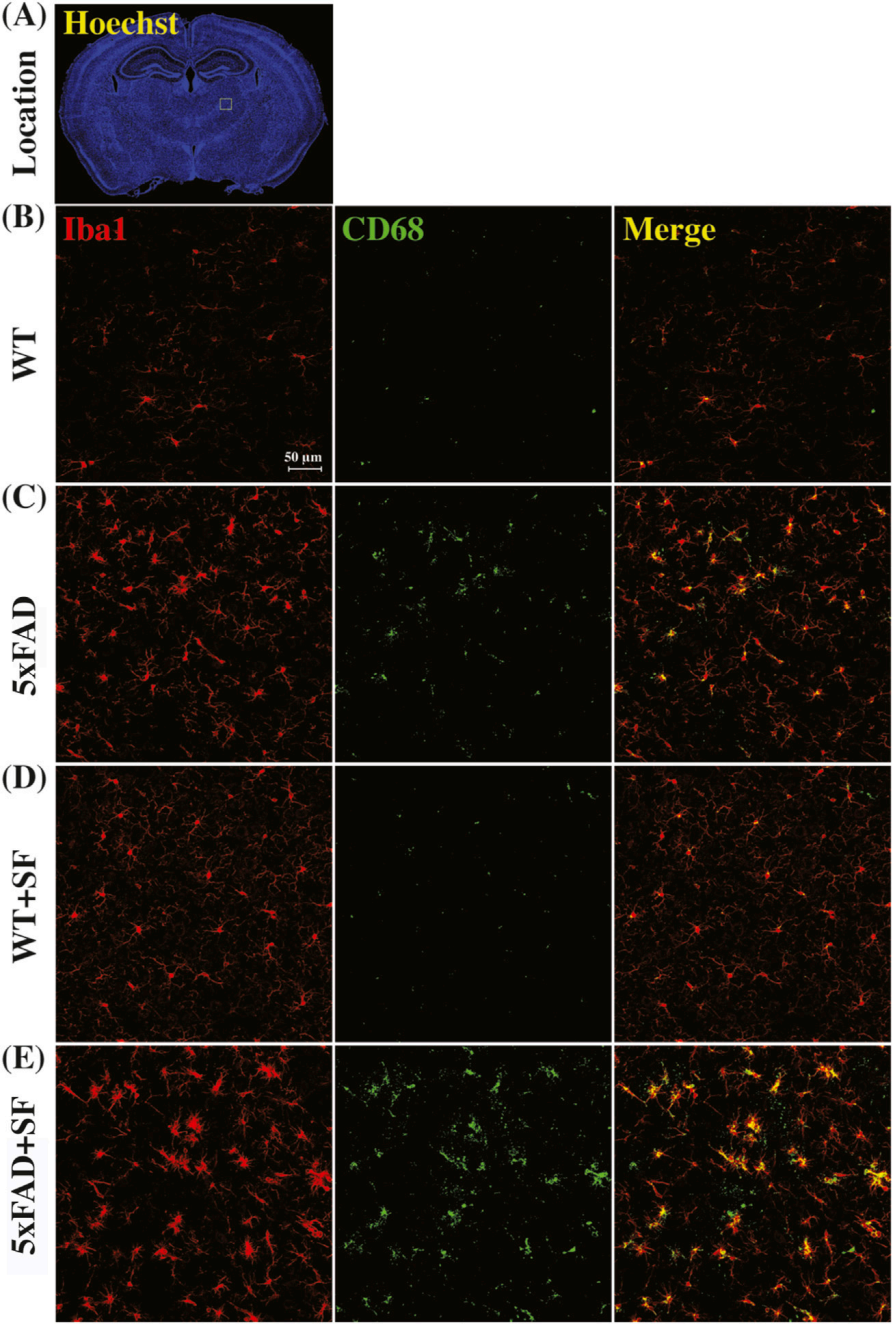
Immunofluorescence staining of neuroinflammation at the ×20 magnification. **(A)** Presents the Hoechst staining focusing on nuclei to demonstrate the observation window (square outlined in the right thalamus region) at the ×20 magnification. Representative images of WT **(B)**, 5xFAD **(C)**, WT + SF **(D)**, and 5xFAD + SF **(E)** were presented. Red: Iba1; green: CD68; yellow spots in the merged images denote the activated microglia.

**FIGURE 5:**
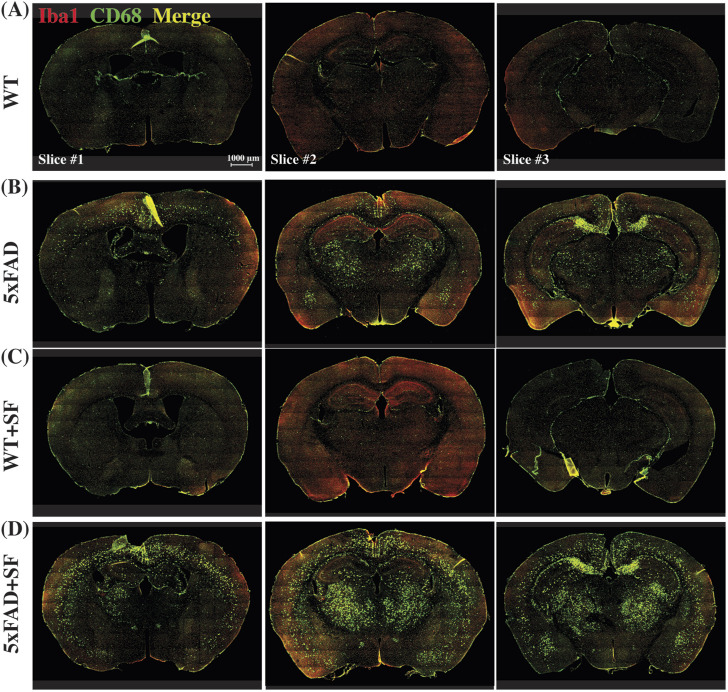
Immunofluorescence staining of neuroinflammation at the ×10 magnification. Representative mice were presented for WT **(A)**, 5xFAD **(B)**, WT + SF **(C)**, and 5xFAD + SF **(D)**. Three slices (#1, #2, and #3) were stained for each mouse. Red: Iba1; green: CD68; yellow spots in the merged image denote the activated microglia.

**FIGURE 6:**
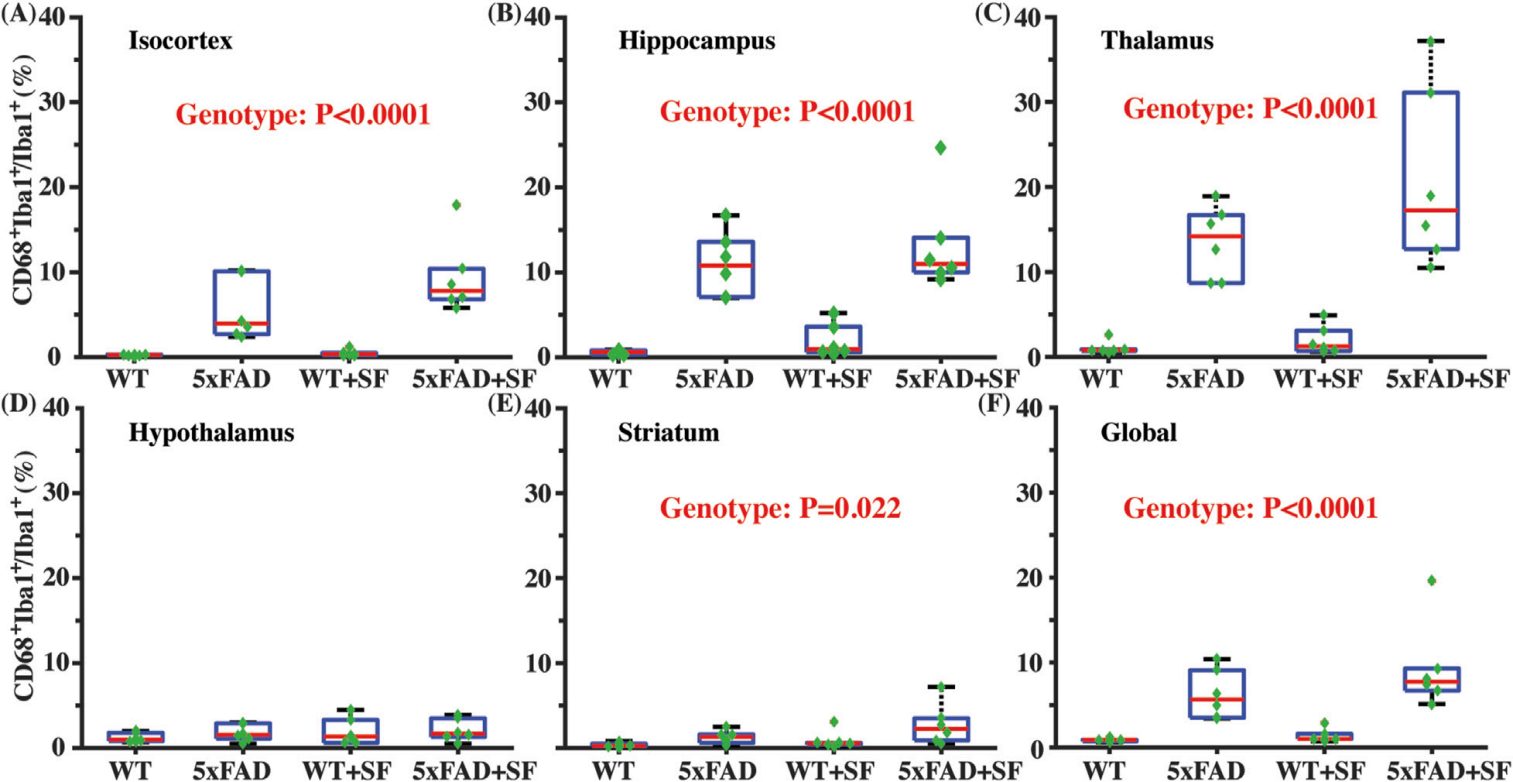
Neuroinflammatory profiles in 5xFAD complicated by SF. Comparisons of activated microglia in isocortex **(A)**, hippocampus **(B)**, thalamus **(C)**, hypothalamus **(D)**, striatum **(E)**, and whole slices **(F)**.

Figure 4 presents the immunofluorescence staining results at ×20 magnification to demonstrate the overall staining quality. A small region in the thalamus (outlined by the square at the right thalamus in Figure 4A) was focused in representative brains. CD68-positive and Iba1-positive signals co-localized well in each group (Figures 4B–E). Figure 5 shows whole-slice microscope images of representative mice for the four experimental groups. Profound microglial activations were observed in the 5xFAD and 5xFAD + SF mice (Figures 5B, D) in comparison with the WT and WT + SF mice (Figures 5A, C). Moreover, the microglial activation arose in both isocortex and deep brain regions. After regional analyses on brain slices of all mice (72 slices totally), we summarized the neuroinflammation levels, which were represented by the percentage of CD68^+^Iba1^+^ cells in relative to total Iba1^+^ cells, as shown in Figure 6. There was not a significant sleep effect (*P* ≥ 0.08) in any of the investigated regions (isocortex, hippocampus, thalamus, hypothalamus, striatum, and whole slice). Genotype effect was significant for isocortex (coefficient = 7.13%, CI = [4.61, 9.66], *P* < 0.0001; Figure 6A), hippocampus (coefficient = 10.90%, CI = [7.89, 13.91], *P* < 0.0001; Figure 6B), thalamus (coefficient = 15.78%, CI = [10.70, 20.85], *P* < 0.0001; Figure 6C), striatum (coefficient = 1.40%, CI = [0.22, 2.58], *P* = 0.022; Figure 6E), and whole slices (coefficient = 6.71, CI = [4.14, 9.27], *P* < 0.0001; Figure 6F), but not for hypothalamus (*P* = 0.47; Figure 6D). The genotype × sleep effect was insignificant (*P* ≥ 0.14) for all the investigated regions. Additionally, there was not a significant correlation between perfusion level and neuroinflammation in any of the tested regions (Pearson correlation, *P* ≥ 0.16).

## 4 Discussion

In this study, we identified imaging markers of cerebral metabolism and perfusion to investigate functional alterations in the co-pathogenesis of AD and SF in the 5xFAD mouse model. Our imaging observations suggest that SF can affect brain metabolism and cerebral perfusion in both WT and 5xFAD mice. Brain metabolism consistently increased following SF. In contrast, cerebral perfusion was differentially affected by SF in WT and 5xFAD mice. Neuroinflammation is primarily influenced by genotype rather than SF in 5xFAD mice at 4 months of age. These results support the use of metabolic and vascular imaging markers to advance future studies on the pathophysiology of AD complicated by SF.

Our study indicates that SF is associated with increased OEF and CMRO_2_. This finding is consistent with a previous human study reporting increased carbohydrate oxidation (Hursel et al., 2011) and a mouse study reporting incremented glucose metabolism (Ba et al., 2021) following SF since anaerobic metabolism is the primary source of energy supply. This metabolic increase may be attributed to the increased activity of sympathetic nervous system because SF can shift the sympathovagal balance (Stamatakis and Punjabi, 2010). Normal aging can lead to hypermetabolism for compensating neuronal deficiency (Lu et al., 2011). Consequently, the aged population are more vulnerable to developing metabolic abnormalities following SF because their vascular reserves may have already been consumed (Peng et al., 2018) to compensate degenerated vascular function and thereafter maintain a normal CBF level to meet the metabolic requirement. The development of vascular degeneration, e.g., stenosis, loss of smooth muscle cell, or basement membrane thickening, is progressive in normal aging. By contrast, patients with vascular problems, e.g., small vessel disease, suffer from additional vascular impairment, and are therefore vulnerable population to develop metabolic abnormalities after SF. Regarding AD cases with amyloidosis, cerebral amyloid angiopathy (CAA) gradually forms to degenerate the vascular function and reduce blood supply (Maier et al., 2014). The SF-induced hypermetabolism further exacerbate this blood-supply deficiency. Consequently, there is an increasing risk of hypoxia, which will promote the Aβ peptide formation and aggregation (Hassan and Chen, 2021).

Sleep is closely associated with the cerebrospinal fluid (CSF) clearance. Dyes injected into the CSF barely flow when the mice are awake, but flow rapidly when the mice are asleep, suggesting that the sleeping state is linked to higher CSF clearance activity (Xie et al., 2013). This is possibly because there are a series of channels expanding during sleep to allow for more extracellular volume to promote the convective flow between CSF and interstitial fluid (Xie et al., 2013; Mendelsohn and Larrick, 2013). SF will induce frequent arousals in the sleep cycle to interrupt the CSF clearance function. The increased metabolism after SF, which eventually generates more metabolic waste, further challenges the CSF clearance function. Consequently, there is a risk of metabolic waste accumulation, among which reactive oxygen species (ROS) can induce impairment to synaptic plasticity and neuronal function (Massaad and Klann, 2011). In complication with AD, oxidative stress plays a vital role to modulate the AD pathophysiology (Manoharan et al., 2016).

**FIGURE 7:**
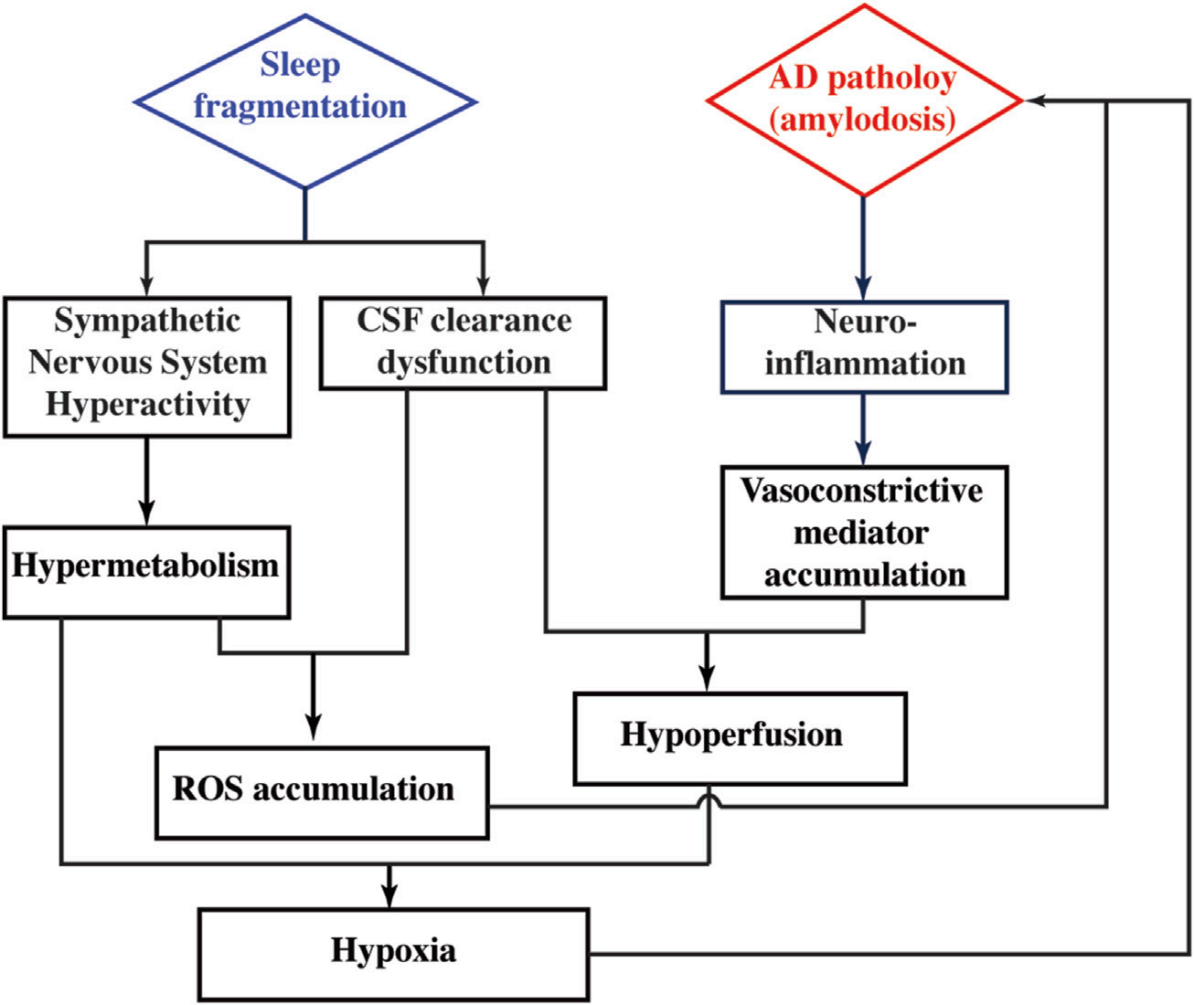
Hypothetical model to explain the pathophysiological alterations in 5xFAD complicated by SF.

Regional perfusion measurement reveals that CBF is differentially affected by SF in WT and 5xFAD. This sleep × genotype interaction effect is more prominent in deep brains of hippocampus and thalamus. In WT mice, CBF values without and with SF are 269.2 ± 47.3 and 317.5 ± 43.2 mL/100 g/min, respectively, suggesting that SF induces a CBF increase in WT. In a human study employing healthy volunteers, increased CBF is found after a day of sleep deprivation (Elvsåshagen et al., 2019), indicating that CBF increase is a common response after sleep perturbation. Taking the metabolic alteration into consideration, CBF increase in WT after SF is possibly a response to the increased activity of sympathetic nerve system. On the other hand, CBF is reduced from 302.5 ± 30.7 to 273.5 ± 44.5 mL/100 g/min in 5xFAD mice following SF. The lack of a CBF increase in 5xFAD + SF mice may be attributed to the interplay between neuroinflammation and CSF clearance. Neuroinflammation is known to have higher expression of pro-inflammatory cytokines, which are capable of activating signaling pathways to modify the activity of vasoconstrictive mediators (e.g., endothelin and angiotensin II) (Sprague and Khalil, 2009; Sankar et al., 2019). The 5xFAD mice tend to secret more vasoconstrictive mediators due to significantly higher levels of neuroinflammation. Normally, these mediators can be cleared from brain by the CSF flow. However, with the presence of SF, CSF clearance is disrupted in 5xFAD mice, leading to an accumulation of vasoconstrictive mediators in the brain and leading to a CBF decline. Consequently, OEF change is more prominent in 5xFAD (42.2%) than that in WT (11.5%) after exposure to SF. Based on our findings and literature reports discussed above, we have summarized a hypothetical model in Figure 7 to explain the metabolic and vascular alterations in 5xFAD complicated by SF.

A prominent and consistent genotype effect on the neuroinflammation was observed. Our observation that SF does not aggravate neuroinflammation is consistent with a human study reporting unchanged markers of systemic inflammation after SF (Stamatakis and Punjabi, 2010). By contrast, a rat study with 72 h sleep deprivation, which is a more acute sleep disruption, reports a recoverable elevation of neuroinflammatory markers (Suresh et al., 2021). These results may indicate that neuroinflammation alteration depends on the severity of sleep disruption. Moreover, mice are tested at a young age in this study due to the intention to provide early imaging markers. Future studies utilizing more aggressive sleep manipulation in older mice will shed more light upon the role of sleep disruption on neuroinflammation during the AD pathology. In literature, mRNA expression of Iba1 is unaltered at 3 months of age in 5xFAD mice (Manji et al., 2019). At the 4 months of age in 5xFAD mice, Iba1^+^ cell area is significantly elevated in hippocampus but not cortex, exhibiting a heterogenous spatial pattern (Forner et al., 2021). In this study, we found that the activated microglia were associated with a significant genotype effect in 4-month-old 5xFAD mice. Collectively, it appears that 5xFAD mice experience more severe neuroinflammation at 4 months of age compared to 3 months, suggesting that 3- to 4-month period may be a turning point for the neuroinflammation cascade. The inconsistent finding in neuroinflammation in the cortex at 4 months of age may be attributed to intersubject variability in disease progression due to environmental factors specific to the local animal facility (e.g., normal diets from different vendors may have slightly different ingredients).

Relationship between neuroinflammation and amyloid plaque deposition in AD has been actively investigated in literature. Inflammation-related protein shows co-localization with amyloid plaque in 5xFAD mice, indicating a potential mechanistic relationship between neuroinflammation and Aβ deposition (Ardestani et al., 2017). This finding is consistent with a human study where MCI cases with low baseline but subsequently rising Aβ load show correlated levels of microglial activation (Ismail et al., 2020). Aβ serves as an initial inflammatory stimulus to trigger activation of the resident microglia followed by increased secretion of various proinflammatory cytokines to recruit further microglia and astrocytes to the inflammatory site (Minter et al., 2016; Fruhwürth et al., 2024). The normal processes of immune cell recruitment, pathogen removal, and inflammatory response resolution are perturbed by the AD pathology (Minter et al., 2016). Cellular contents and damage-associated molecular patterns released by degenerating neurons triggers deleterious microglial reactivity (Heneka et al., 2015; Latta et al., 2015). With the developing neuroinflammatory environment, pro-inflammatory cytokine secretion is dysregulated and neurodegeneration is exacerbated (Sudduth et al., 2013). Based on the literature, it is evident that neuroinflammation and Aβ deposition interact with and influence each other.

Brain physiology is an active field providing a rich source of promising biomarkers for studying normal aging and neuronal diseases. In this study, we showed the potential of brain perfusion and metabolism measurements for studying the co-pathogenesis between AD and SF. The MRI techniques utilized in current study have already been broadly optimized for human study and are implementable at clinical scanners (Lu et al., 2011; Peng et al., 2015; Jiang et al., 2019), indicating that the presented MRI-based physiological measurements are associated with high clinical translational value.

Post-hoc data power calculations were performed with Monte Carlo simulation (2,000 iterations) based on the collected data. Regarding the imaging findings, data powers were 0.77, 0.72, and 0.75 for the sleep effect in OEF, interaction effect in CBF, and sleep effect in CMRO_2_, respectively. In the histology analyses, data powers were 0.99, 0.99, 0.99, 0.71, and 0.99 to observe the genotype effect on activated microglia in the isocortex, hippocampus, thalamus, striatum, and whole brain, respectively.

Findings in current study should be interpreted in the context of several limitations. First, an established experimentally induced SF model instead of age-related SF was employed because the current study aimed to provide potential early imaging markers, while age-related SF occurred at relatively advanced ages (Miner and Kryger, 2017). There could be differences between the experimentally induced SF and age-related SF. Environmental factor (e.g., noise, light), mental status (e.g., stress, depression), diet (e.g., alcohol, caffeine), and circadian misalignment are commons reasons of SF (Potter et al., 2016; Binks et al., 2020). These risk or causal factors may directly affect AD independently of SF (Killin et al., 2016). Second, oxygen consumption was measured in a global manner without spatial information. Regional maps will further enhance the understanding of metabolic heterogeneity across brain regions. Thirdly, this study has only used male mice. Potential sex effects require further investigations. Meanwhile, the present study is a proof-of-principle demonstration to show the potential of using non-invasive imaging techniques to facilitate the understanding of co-pathogenesis of AD and SF. Despite of the effort to propose a hypothetical model reconciling our current findings and literature reports, systematic mechanistic studies are still required to confirm these hypothetical explanations. Meanwhile, longitudinal MRI tracking of the metabolic and vascular abnormality is desired to reveal the onset and progression of different microvascular dysfunctions. Neuroinflammation was examined with brain sections but not blood samples. It would be useful to evaluate the expression of serum Aβ1-42, Aβ1-40, and inflammatory factors such as TNFα, IL-6, and IL-1β in future studies. Finally, beyond the scope of physiological functions, further characterizations using metabolomics (Lin et al., 2017; Lin C. et al., 2019) or chemical exchange saturation transfer (CEST) (Han et al., 2022; Lee et al., 2019) will be helpful in providing a molecular-level screening and cross-validating the current findings.

## 5 Conclusion

In this proof-of-principle study, we employed non-invasive, non-contrast MRI techniques to examine functional alterations in an amyloidosis AD mouse model with sleep disruption. Our results demonstrate that MRI techniques are sensitive to the metabolic and vascular dysfunctions associated with the co-pathogenesis of AD and SF. These findings support the use of cerebral perfusion and brain metabolism as promising imaging markers for investigating the pathophysiology of AD complicated by sleep disruption.

## Data Availability

The raw data supporting the conclusions of this article will be made available by the authors, without undue reservation.
